# DAF-2/Insulin-Like Signaling in *C. elegans* Modifies Effects of Dietary Restriction and Nutrient Stress on Aging, Stress and Growth

**DOI:** 10.1371/journal.pone.0001240

**Published:** 2007-11-28

**Authors:** Wendy B. Iser, Catherine A. Wolkow

**Affiliations:** Laboratory of Neurosciences, National Institute on Aging Intramural Research Program, National Institutes of Health, Baltimore, Maryland, United States of America; Duke University Medical Center, United States of America

## Abstract

**Background:**

Dietary restriction (DR) and reduced insulin/IGF-I-like signaling (IIS) are two regimens that promote longevity in a variety of organisms. Genetic analysis in *C. elegans* nematodes has shown that DR and IIS couple to distinct cellular signaling pathways. However, it is not known whether these pathways ultimately converge on overlapping or distinct targets to extend lifespan.

**Principal Findings:**

We investigated this question by examining additional effects of DR in wildtype animals and in *daf-2* mutants with either moderate or severe IIS deficits. Surprisingly, DR and IIS had opposing effects on these physiological processes. First, DR induced a stress-related change in intestinal vesicle trafficking, termed the FIRE response, which was suppressed in *daf-2* mutants. Second, DR did not strongly affect expression of a *daf-2*- and stress-responsive transcriptional reporter. Finally, DR-related growth impairment was suppressed in *daf-2* mutants.

**Conclusions:**

These findings reveal that an important biological function of DAF-2/IIS is to enhance growth and survival under nutrient-limited conditions. However, we also discovered that levels of DAF-2 pathway activity modified the effects of DR on longevity. Thus, while DR and IIS clearly affect lifespan through independent targets, there may also be some prolongevity targets that are convergently regulated by these pathways.

## Introduction

Two pathways affecting lifespan in a variety of species are dietary restriction (DR) and insulin/IGF-I-like signaling (IIS). However, there is still debate about how these pathways actually extend lifespan. From genetic studies in *C. elegans*, it is known that DR and IIS couple to distinct cellular signaling pathways. For example, the *daf-16* gene, which encodes a FOXO transcription factor, is absolutely essential for longevity in IIS mutants, but is dispensable for long lifespan from DR [1]–[3]. Furthermore, DR can further lengthen lifespan of long-lived animals lacking IIS due to a mutation in the kinase domain of the DAF-2/insulin/IGF-I receptor (IR) [1]–[3]. These genetic data argue that the DAF-2/insulin-like pathway is not required in cells to transduce the effects of DR.

However, these studies do not exclude the possibility that DR and IIS converge on downstream targets that act to promote longevity. Indeed, there is some evidence supporting this possibility. In *C. elegans,* certain superoxide dismutase (*sod*) genes are specific targets of either DR or IIS, while other *sod* genes are targets of both pathways [4]. In *Drosophila*, evidence for converging DR and IIS outputs was suggested by observations that DR sensitivity was altered in *chico* mutants, which have prolonged lifespan from disrupted IIS [5]. However, further work is needed to fully understand the interactions between these pathways.

One approach to deciphering the relationship between DR and IIS is to compare their effects on physiological processes including, but not limited to, lifespan. A more thorough examination of interactions between DR and IIS would provide additional insight into the effects of each pathway. This approach is possible in the nematode, *C. elegans*, where interactions between DR and IIS can be examined in double mutants with lesions affecting each pathway [6]. Furthermore, the effects of titrating IIS can be determined by conducting these studies in class 1 or class 2 *daf-2* mutants. Class 1 and 2 *daf-2* mutations were previously defined by their phenotypic severity [7]. Both classes cause dauer larval arrest, adult longevity and stress resistance, but class 2 mutants exhibit additional phenotypes not seen in class 1 mutants, such as reduced fertility and movement.

Using the collection of experimental reagents available to study DR and IIS in *C. elegans*, we have studied physiological effects of DR in both wildtype and IIS-deficient *daf-2* mutants. In particular, this analysis focused on two cellular stress responses easily visualized in *C. elegans*. One is the fasting-induced redistribution of esterase activity in intestinal cells, referred to as the FIRE assay (Fasting-Induced Redistribution of Esterase activity). In addition, *C. elegans* cells also induce expression of heat-shock proteins, such as *hsp-16.2*, in response to stressful stimuli, such as high temperature. The data suggest that DR and IIS regulate distinct targets to affect cell stress and body size. More careful characterization of DR's effects on lifespan of *daf-2* mutants revealed that the level of *daf-2* activity modulates the prolongevity effects of DR. This unexpected finding provides new evidence that IIS may extend lifespan by multiple mechanisms, some of which overlap with those affected under DR conditions.

## Results

### DR induces the FIRE response to cellular stress

**Figure 1:**
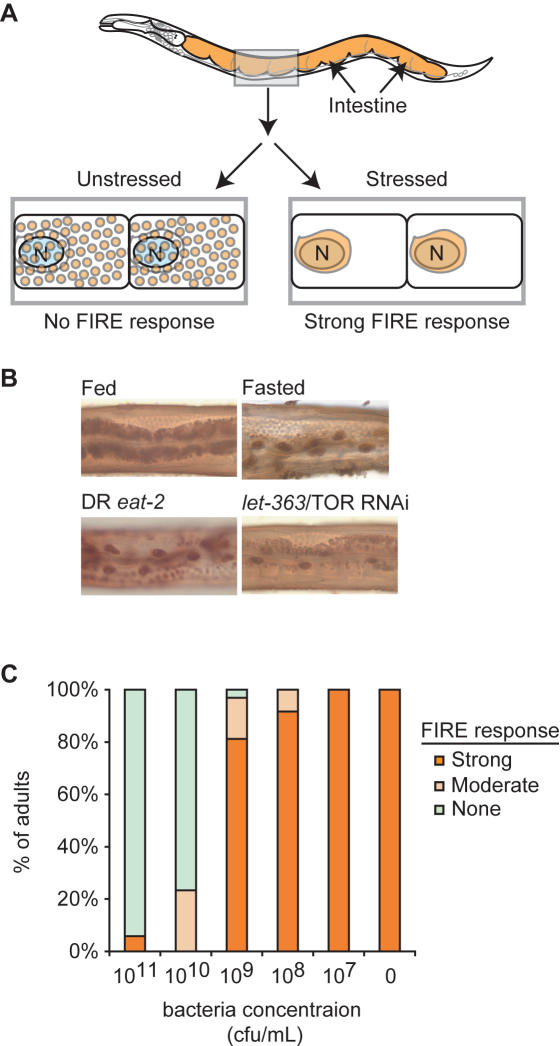
Dietary restriction (DR) induces the FIRE response to cellular stress in *C. elegans* intestinal cells. (A) Cartoon of the FIRE response depicting the stress-induced redistribution of cytoplasmic esterase activity to a perinuclear region. The large rectangles contain cartoons of esterase activity (brown color) localization in two adjacent intestinal cells (rounded rectangles) with nuclei indicated by ovals labeled “N”. (B) Cytoplasmic esterase activity in intestinal cells of young adult hermaphrodites in the presence of ample bacterial food (Fed), 5-hours following food withdrawal (Fasted), and in *eat-2(ad465)* mutants (DR *eat-2*) or wildtype animals subjected to RNAi knockdown of *let-363*/TOR (*let-363*/TOR RNAi). Images are representative from 80 or more animals examined in at least 3 independent experiments. (C) DR induced by food source dilution also induced a FIRE response in wildtype adults. Young adult hermaphrodites, raised under bacteria-replete conditions, were fed with bacteria at indicated concentration for 5 hours and then analyzed. At high food concentrations (10^10^ and 10^11^ cfu/mL) adult animals did not exhibit a FIRE response. At lower food concentrations, (<10^9^ cfu/mL), most animals exhibited a strong FIRE response. For each feeding condition, FIRE response was scored in 17-32 animals in a blinded fashion. Similar results were obtained in 2 additional experiments using a progressive nutrient stress regimen of diluted *let-363*/TOR RNAi (not shown).

As a first step to studying interactions between DR and DAF-2/IIS, we examined the effects of these pathways on a marker of cellular stress. Our previous studies of cellular stress responses in *C. elegans* identified a cellular response that is easily visualized in *C. elegans* intestinal cells [8], [9]. This response, termed FIRE for *F*asting-*I*nduced *R*edistribution of *E*sterase, is the progressive relocalization of intestinal esterase activity from the cytoplasm to the nucleus upon fasting (Fig. 1A). The FIRE response was reversible, as esterase activity became completely cytoplasmic within hours of refeeding (not shown). The FIRE response was not restricted to nutrient stress, as it could be triggered by high temperature and oxidative stress (not shown). We hypothesize that the FIRE response reflects a stress-induced alteration in cellular trafficking pathways that leads to accumulation of vesicular cargo, such as the intestinal esterase enzymes, in a nuclear or perinuclear compartment.

The original procedure for inducing the FIRE response involved complete removal of food from adult animals that had matured under food-replete conditions [9]. A similar regimen, termed dietary deprivation (DD), was shown to increase lifespan and stress resistance [10], [11]. Since DD could both prolong lifespan and induce the FIRE response, we investigated whether DR regimens reducing, but not eliminating, food intake could also induce a strong FIRE response. Indeed, two different approaches for DR did induce the FIRE response in adult animals. First, genetic mutations causing DR induced a robust FIRE response. A robust FIRE response was observed in *eat-2(ad465)* animals, which are subject to chronic DR from reduced food intake, and in animals undergoing RNAi knockdown of *let-363*, which encodes the TOR kinase essential for cellular nutrient signaling (Fig. 1B) [3], [12], [13]. Furthermore, a robust FIRE response was detected in animals subject to DR as a consequence of low food availability (Fig. 1C). In particular, dilution of the bacterial food source to 10^9^ cfu/mL induced a strong FIRE response in most animals. This bacterial concentration is within the range of concentrations that were previously shown to promote longevity by DR [2].

### IIS mutations promote resistance to DR-induced FIRE response

**Figure 2:**
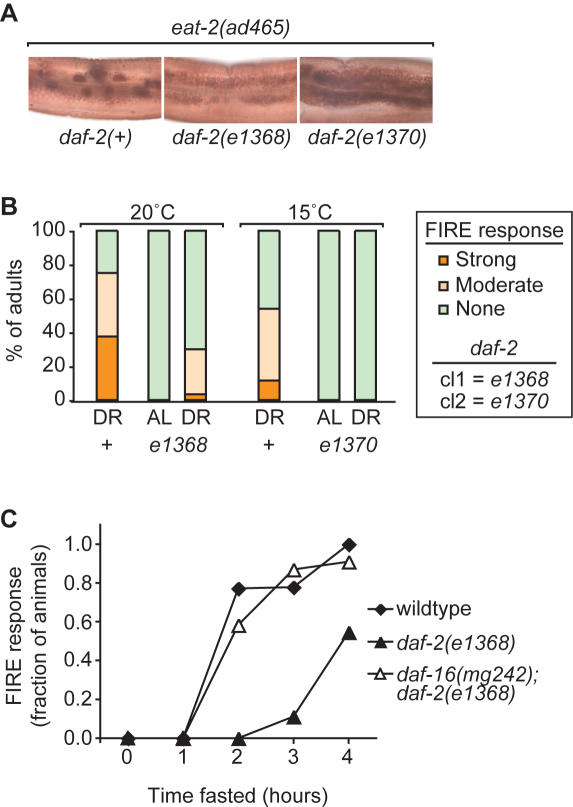
* daf-2* mutations suppressed DR-induced FIRE response. (A, B) Representative images showing FIRE response induction in *eat-2(ad465)* animals and suppression in two different *eat-2*; *daf-2* double mutants. (B) FIRE response suppression was stronger in *daf-2(e1370)* animals than in *daf-2(e1368)* animals. Quantification of the FIRE response in the same strains shown in (A); n = 22–30 animals examined per strain, results are from one representative of three or more experiments. Assays were conducted at 20°C for *daf-2(e1368)* and relevant controls or at 15°C for *daf-2(e1370)* and controls. (C) *daf-16* activity is required for resistance to FIRE-inducing conditions in *daf-2* mutants but not wildtype animals. Graph shows the fraction of animals displaying a strong FIRE response during a time course of fasting; wildtype (diamonds), *daf-16(mg242); daf-2(e1368)* (open triangles) and *daf-2(e1368)* (filled triangles). Results are for one representative of 4 experiments; n = 7–23 animals (average, 16 animals) examined per time point per strain; total number of animals examined per strain, wildtype (102 animals), *daf-2(e1368)* (65 animals), *daf-16(mg242); daf-2(e1368)* (72 animals). Consistent results were also observed after *let-363*/TOR RNAi.

Mutations disrupting IIS in *C. elegans* are correlated with increased resistance to a spectrum of environmental stresses, including high temperature, oxidative stress and ionizing radiation. These stresses trigger the nuclear localization and activation of DAF-16/FOXO [14]–[16]. Food deprivation also triggered DAF-16/FOXO nuclear localization suggesting that nutrient stress may couple to IIS in *C. elegans* [14], [16]. To further examine the role of IIS in regulating resistance to nutrient stress, we examined whether *daf-2* mutant adults exhibited a normal FIRE response under DR conditions. For these experiments, we constructed double mutants carrying either a class 1 or 2 *daf-2* mutation along with the *eat-2(ad465)* mutation, to induce DR. Class 1 and 2 *daf-2* mutations were previously defined by their phenotypic severity [7]. Both classes cause dauer larval arrest, adult longevity and stress resistance, but class 2 mutants exhibit additional phenotypes not seen in class 1 mutants, such as reduced fertility and movement. We examined the effects of DR in animals carrying either the *e1368* (class 1) allele mutated in the DAF-2/IR ligand-binding domain, or the *e1370* (class 2) allele mutated in the DAF-2/IR kinase domain [17]–[19]. Both the *e1368* and *e1370* alleles could effectively suppress the FIRE response associated with DR (Fig. 2A, B). However, FIRE response suppression was stronger in animals with the class 2 *e1370* allele than in animals with the class 1 *e1368* allele. This finding suggests that the level of IIS disruption was correlated with the level of resistance to DR-induced cellular stress.

The major output of DAF-2 signaling in *C. elegans* is the DAF-16/FOXO transcription factor [20], [21]. Mutations in *daf-16* suppress adult longevity and stress resistance phenotypes in *daf-2* mutants [22], [23]. In addition, *daf-16* mutations suppress the FIRE-response resistance of *daf-2* mutants under nutrient deprivation (Fig. 2C) [8]. For some other stresses, such as aging, oxidative stress and temperature, *daf-16* mutants exhibit increased sensitivity compared to wildtype animals, suggesting that these stresses trigger DAF-16 activation in wildtype animals [8], [16], [24]. To determine whether *daf-16* activity was required for a normal FIRE response in wildtype animals, we compared the kinetics of FIRE response induction in wildtype animals and *daf-16* mutants. As expected, the FIRE response was delayed in *daf-2(e1368)* animals compared to wildtype (Fig. 2C). In double mutant *daf-16(mg242); daf-2(e1368)* animals, the FIRE response was restored and was not noticeably different from that in wildtype animals (Fig. 2C). We take this evidence that DAF-16 function is not required for a normal FIRE response in wildtype animals under these conditions. Thus, *daf-16* mutants do not display increased sensitivity to the stresses associated with the FIRE response in this context.

### DR selectively affected the FIRE response, but not HSP expression, in *C. elegans*

Because DR induced the FIRE response to stress, we wondered if DR was a generally stressful condition that induced multiple stress response pathways. We examined expression of two *C. elegans* stress-inducible GFP reporters expressed from the promoters for *hsp-16.2* or *gst-4*. Both the *hsp-16.2*:GFP and *gst-4*:GFP reporters have been shown to respond to oxidative and/or thermal stress [25], [26].

**Figure 3:**
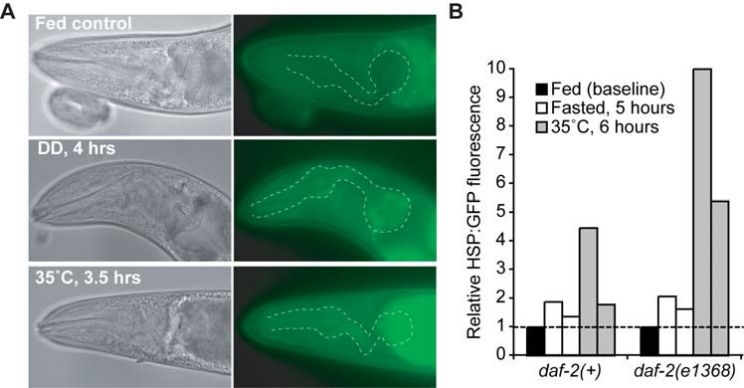
Nutrient stress did not strongly induce *hsp-16.2:GFP* reporter expression. A GFP reporter expressed from the *hsp16.2* promoter was examined in animals under food-replete, fasted and heat-stressed conditions. (A) Under food-replete conditions (fed), fluorescence from *hsp16.2:*GFP was undetectable in the pharynx region of most animals. After a 4 hour fasting regimen, elevated *hsp-16.2:*GFP fluorescence was observed in many animals, as it was for animals under thermal stress (35°C, 3.5–6 hours). (B) Relative GFP fluorescence in the pharynx region of animals carrying the *hsp-16.2:GFP* reporter. Fluorescence intensity was measured as average pixel intensity in same-sized regions of the pharynx. Fluorescence intensity measurements under food-replete conditions were set as baseline. Duplicate bars indicate results from two independent experiments; n = 14–19 animals scored per condition in each experiment. In both experiments, 35°C heat stress induced a statistically significant increase in *hsp-16.2*:GFP fluorescence intensity (p≤0.001 compared to fed controls). Nutrient stress was not correlated with reproducible statistically significant changes in *hsp-16.2*:GFP fluorescence intensity (p≤0.001 in one trial only). All conditions were tested on the same day and images were collected using identical acquisition parameters. Three additional experiments also gave similar results.

Fluorescence from the *hsp-16.2*-expressed GFP reporter was slightly increased in the pharynx after 5 hours without food, compared with fed controls (Fig. 3) [26]. However, the level of *hsp-16.2*:GFP induction by DR or fasting was significantly lower than the induction level from thermal stress, induced by exposing transgenic animals to the stressful temperature of 35°C for 5 hours (Fig. 3B). A minor increase in *hsp-16.2*:GFP fluorescence was sometimes observed in animals under DR by food dilution, although this response was inconsistent between independent experiments (not shown). Finally, fluorescence from the *hsp-16.2*:GFP reporter was not detectably elevated in *eat-2(ad465)* young adults (not shown). Fasting in *daf-2(e1368)* mutants had a similar effect on *hsp-16.2:*GFP expression, although *hsp-16.2:*GFP induction was enhanced in this background under thermal stress conditions (Fig. 3). This is consistent with previous reports linking HSP induction to the *C. elegans* IIS pathway [27]. Together, these data indicate that, although severe nutrient stress may have a minor effect on HSP levels as monitored by the *hsp-16.2*:GFP reporter, this effect was not fully consistent under DR conditions. This is in agreement with previous genetic data showing that longevity from DR is independent of *hsf-1*, the heat-shock factor that promotes HSP expression under stress [28], [29]. Similarly, expression of the *gst-4*:GFP reporter was also not increased after fasting, suggesting that this stress-inducible gene was also not affected by nutrient stress (not shown). Thus, nutrient stress under the conditions we tested, selectively induced the FIRE response in *C. elegans*.

### Growth deficits from DR conditions are also suppressed by class 2 *daf-2* mutations

**Figure 4:**
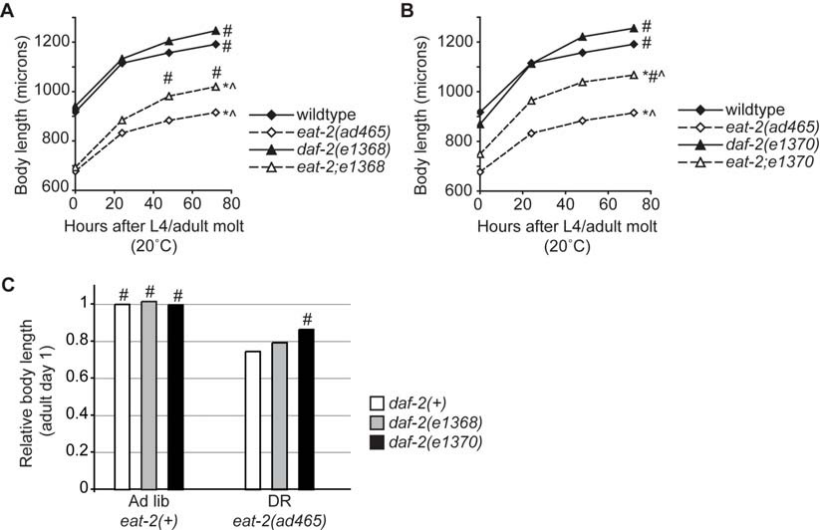
Small body size caused by DR was partially suppressed in *daf-2* mutants. (A, B) Body length increased over the first 3 days of adulthood under both DR and non-DR conditions. Wildtype animals under DR conditions were approximately 30% shorter than under non-DR conditions (diamonds). Under non-DR conditions, *daf-2(e1368)* and *daf-2(e1370)* adults were slightly longer than wildtype animals (dark shapes) and *daf-2* mutant body size was also reduced under DR (triangles). However, *eat-2;daf-2* animals grew to larger sizes than *eat-2* animals (open triangles vs open diamonds). (C) Composite graph of adult body length of wildtype and *daf-2* mutants under DR and non-DR conditions on adult day 1 (24-hours post-molt), plotted relative to wildtype non-DR adults. All body length measurements were conducted at 15°C and animals were synchronized by chronological age from hatching. For each time point, measurements of 10–14 animals for each strain were averaged from 3 independent experiments, except for *eat-2(ad465); daf-2(e1368)* animals for which 2 experiments were conducted. Statistical significance was evaluated by t-test and indicate significance in at least 3 of 4 timepoints, *p<0.01 vs wildtype in all trials, #p<0.01 vs *eat-2(ad465)* in all trials, ˆp<0.01 vs *daf-2(e1368)* (A) or *daf-2(e1370)* (B) in all trials ; p<0.0001 (t-test) for all 3 strains at each time point except for *eat-2(ad465)* vs *eat-2(ad465); daf-2(e1370)* at 0 and 8 hours (p = 0.076 and 0.089, respectively). In addition, in (A) *eat-2(ad465); daf-2(e1368)* vs *eat-2(ad465)* body lengths were statistically significant (p<0.01) at only the 48 and 72 hour timepoints of all trials.

In *C. elegans*, DR regimens can result in short body length, possibly due to reduced protein translation [30]–[32]. In contrast, *daf-2* mutants are similar in size, or slightly longer, than wildtype animals [33]. We therefore examined whether the growth defects of DR were altered in *daf-2* mutants. Measurement of head-to-tail body length confirmed that *eat-2(ad465)* adults were smaller in size than wildtype adults at the same developmental age (Fig. 4). Over the 72-hour period following the final larval molt, the body length of both wildtype and *eat-2(ad465)* adults increased by 25–30%, reflecting growth during early adulthood (Fig. 4) [34]. However, throughout this period, *eat-2(ad465)* adults remained approximately 25% shorter overall than wildtype animals. Similarly, *eat-2; daf-2* double mutants were shorter than *daf-2* single mutants, showing that DR also shortened body length in *daf-2* mutants. However, *daf-2* mutants exhibited substantially more growth under DR conditions than *daf-2(+)* animals. In particular, *eat-2(ad465); daf-2(e1370)* adults were between 11–58% longer than *eat-2(ad465)* adults of over the first 3 days of adulthood (Fig 4B). The *eat-2(ad465); daf-2(e1368)* adults were also longer than *eat-2(ad465)* single mutants, although the effect was less dramatic than for *daf-2(e1370)*. Although both the *daf-2(e1368)* and *e1370* mutants were slightly longer than wildtype animals under non-DR conditions, these differences were not as dramatic as for DR conditions, suggesting that DR enhanced the growth-promoting effect of *daf-2* mutations [33]. The larger body size in *eat-2(ad465);daf-2(e1370)* adults was not due to suppression of the pharynx pumping defect caused by the *eat-2(ad465)* mutation. The pump rate of *eat-2(ad465); daf-2(e1370)* double mutants was indistinguishable from that of *eat-2(ad465)* animals (not shown).

### DR 's effect on longevity differs for class 1 and 2 *daf-2* mutants

**Figure 5:**
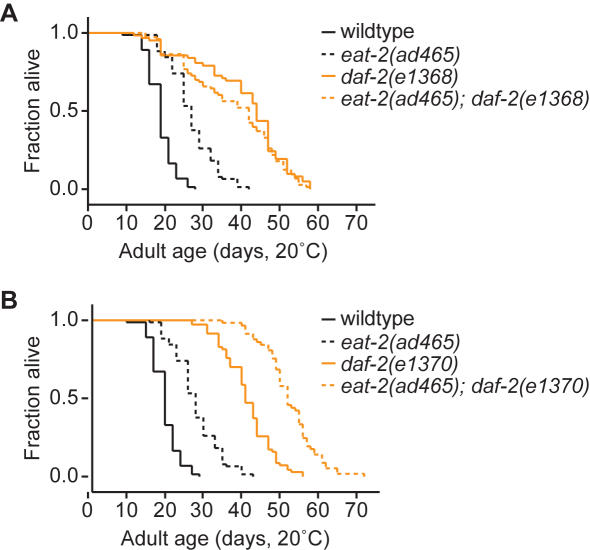
Levels of *daf-2* activity modify lifespan of *eat-2(ad465)* animals. DR, from the *eat-2(ad465)* mutation, had an additive effect on lifespan of *daf-2(e1370)* adults (B), but not on lifespan of *daf-2(e1368)* adults (A). Lifespan data and statistics are presented in Table 1.

Several studies have confirmed that lifespan of class 2 *daf-2(e1370)* adults is lengthened by DR from either food dilution or in *eat-2* mutants [2], [3]. Since class 1 and 2 *daf-2* mutants differed in the ability to suppress the FIRE response and body length phenotypes of DR, we examined whether DR had different effects on lifespan of each mutant. Consistent with previous reports, we observed that *eat-2(ad465); daf-2(e1370)* adults lived substantially longer than *daf-2(e1370)* animals under non-DR conditions (Fig. 5B). This supports with the idea that *e1370* and DR affect lifespan through distinct mechanisms that have additive effects. Surprisingly, DR had no effect on lifespan of *daf-2(e1368)* adults (Fig. 5A). This finding suggests that DR and *daf-2(e1368)* may extend adult lifespan through overlapping mechanisms that are not additive. We noted that lifespan was similar in the *e1368* and *e1370* strains under non-DR conditions (Fig. 5, Table 1). We hypothesize that the *e1368* and *e1370* mutations may alter distinct spectrums of IIS functions that differentially affect survival under DR.

**Table 1:** DR, imposed by the *eat-2(ad465)* mutation, extended adult lifespan of *daf-2(e1370)*, but not *daf-2(e1368)*.

Genotype	Average adult lifespan days (n)		% change	*p* (Log-rank) DR vs non-DR
	Ad lib *(eat-2(+)*)	DR (*eat-2(ad465)*)		
*daf-2(+)*	18.9 (73)	27.0 (77)	+43%	<0.0001
*daf-2(e1370)*	40.5 (70) *	51.4 (57)	+27%	<0.0001
*daf-2(e1368)*	40.5 (62) *	37.9 (73)	−6%	0.19

Adult lifespan was examined at 20°C in the presence of FUDR. Data are cumulative of two trails with 30–40 animals/trial. Two additional trials were conducted for ad lib and DR *daf-2(e1370)* and wildtype animals with consistent results (not shown).

* Non-DR lifespans of *daf-2(e1370)* versus *daf-2(e1368)* were statistically significantly different by the Log-rank test (p = 0.0058) but not by Wilcoxon test p = 0.11), although they were not dramatically different.

## Discussion

Here, we examined interactions between IIS and nutrient stress/DR signaling in *C. elegans.* The goal of this work was to investigate whether DR and IIS couple to overlapping or distinct downstream targets by determining their combinatorial effects on several physiological processes, specifically stress responses, growth and longevity. We reasoned that additional information about interactions between DR and IIS in these processes would be useful for understanding whether these pathways regulate lifespan through overlapping or independent targets.

**Figure 6:**
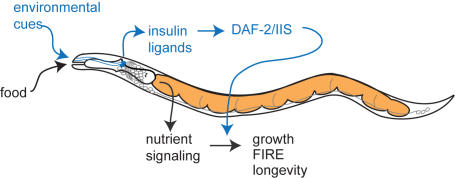
Model for the interactions between DR and DAF-2/IIS on growth, FIRE response and lifespan. Food intake and nutrient signaling have dramatic effects on growth, FIRE response and longevity in *C. elegans*. This paper reports that levels of DAF-2/IIS can modify these three nutrient-dependent processes. We propose that levels of DAF-2/IIS may be under control of environmental cues that modulate insulin ligand production.

Taken together, our findings reveal important new insights regarding the targets of DR and IIS. First, DR and IIS have opposing effects on some physiological processes, despite their parallel effects on longevity. The FIRE response, *hsp-16.2*:GFP expression and body growth were all differentially affected by DR and *daf-2*. It is unlikely that DR and *daf-2* act on identical downstream targets to oppositely regulate these processes. Thus, a reasonable explanation is that DR and IIS regulate distinct targets that have opposing effects on cellular stress and body size. This finding is in accord with other data supporting independent outputs for the prolongevity effects of DR and IIS [2]–[4]. These data also reveal that modulating IIS by DAF-2 receptor signaling may serve as a mechanism for promoting growth and survival under conditions of nutrient deprivation. A model for these results proposes that nutrient signaling, in response to food intake and availability, regulates the processes involved in growth, FIRE response and longevity (Fig. 6). DAF-2/IIS acts in parallel to the nutrient signaling pathway to modify the organism's growth, FIRE response and longevity under conditions of low food availability. Environmental cues may modulate the level of DAF-2/IIS through effects on insulin-like ligand production. Consistent with this idea, two insulin-like ligands, DAF-28 and INS-1, have been shown to promote responses to environmental cues [35]–[37].

We also found that *daf-2(e1368)* animals did not display any further lifespan increase under DR. In contrast, *daf-2(e1370)* animals lived significantly longer under DR. By conventional genetic analysis, pathways with independent outputs should demonstrate additive or synergistic interactions, except in the case of non-null conditions. However, it is difficult to interpret the differential interactions with DR, since both *daf-2* mutations are hypomorphic, but not null, alleles [7], [17]. This finding may reveal a condition under which DR and IIS convergently regulate common prolongevity targets. Of the two *daf-2* alleles examined, *e1368* was also the least effective in suppressing other DR-induced phenotypes. Thus, we propose that DAF-2 IIS regulates two classes of targets. One target class, activated in both *daf-2(e1368)* and *e1370* animals, promotes longevity and overlaps with at least some DR prolongevity targets. Such co-regulation has been reported for the MnSOD enzyme encoded by *sod-1* in *C. elegans* [4]. The second target class is preferentially affected in *daf-2(e1370)* animals, but not in *e1368* animals, and appear to promote growth and increased survival under nutrient deprivation and DR conditions.

Could there be a connection between DR's effects on cellular stress and longevity? The FIRE response appears to be a marker for cellular stress, as it is induced by a variety of stresses, including nutrient, thermal and oxidative stress. However, unlike thermal stress, nutrient stress did not induce expression of the *hsp-16.2*:GFP reporter. Thus, these nutrient stress conditions selectively activated the FIRE response, but not *hsp-16.2*:GFP expression. DR has been proposed to affect longevity through a process of stress hormesis, where sublethal levels of stress promote beneficial effects [38], [39]. Stress hormesis from thermal and oxidative stresses have the ability to extend *C. elegans* lifespan, showing that stress hormesis pathways are functional in this organism [40]–[42]. However, it is not yet known whether DR extends *C. elegans* lifespan through a stress hormesis-like pathway. Our study is the first to show that DR in *C. elegans* induces mild stress, which selectively induced the FIRE response, thus suggesting that pathways affected during stress hormesis may contribute to the effect of DR on lifespan in this organism. Although nutrient deprivation and DR robustly induced the FIRE response, we did not detect a consistent effect on other stress-responsive reporters. Thus, DR had selective effects on cellular stress responses.

## Materials and Methods

### Strain maintenance and growth

All strains were maintained at 15°C on NGM agar medium on OP50 bacterial lawns for a food source. Strains used in this study were: wildtype (N2, Bristol), CB1370 (*daf-2(e1370)*), DR1572 (*daf-2(e1368)*), DA465 (*eat-2(ad465)*), CL2070 (*dvIs70* carrying *hsp-16.2:GFP*) and CL2166 (*dvIs19* carrying *gst-4:GFP*). The *daf-16(mg242)* allele was crossed into the *daf-2(e1368)* background from the strain WCAW118 (*daf-16(mg242); sqt-1(sc13) age-1(mg109)*) by mating heterozygous WCAW118 males with *daf-2* mutant hermaphrodites and then selecting second generation dauer larvae that were homozygous for the *daf-2* mutation. The dauer larvae were recovered and their progeny screened for mixtures of dauer and non-dauer progeny at 25°C, indicating the presence of the heterozygous *daf-16* mutation. Non-dauer progeny were isolated and cultivated to isolate a homozygous *daf-16* mutant strain. A similar strategy was utilized for crossing the *eat-2(ad465)* mutation into the *daf-2* mutant background, except that recovered F2 dauers were screened for slow pump rates (<60 ppm in *eat-2(ad465)* vs >200 ppm in the *daf-2* mutants at 15°C) to identify animals homozygous for the *eat-2(ad465)* mutation.

### RNA interference by feeding

For synchronous populations, approximately 5 gravid adult hermaphrodites were allowed to deposit eggs for several hours on a lawn of dsRNA-expressing bacteria covering NGM agar supplemented with ampicillin (100 µg/mL) and IPTG (1 mM). Hatchling larvae consumed the dsRNA-expressing bacteria as a food source during development, which induced systemic RNAi to the corresponding gene [43], [44]. In control experiments, we have measured approximately 70% knockdown of transcript levels in animals grown on the corresponding dsRNA-expressing bacteria for several different genes (*rps-1*, *let-60*, *mpk-1*) (S.K.Rao, A. DePina & C.A. Wolkow, unpublished). Thus, we expect that the RNAi feeding protocol used for the current study also likely reduces, but does not eliminate, target gene expression.

### Assays for fasting response, adult lifespan and length

The cellular fasting response, termed FIRE, was assayed as previously reported in young adults after one or two generations of growth on dsRNA-expressing bacteria or without dsRNA treatment, as indicated [8], [9]. Briefly, young adults were washed from growth medium in M9, rinsed and then fixed for 10 minutes in cold methanol (−20°C). After fixation, animals were stained to detect *in situ* esterase activity by incubation overnight at room temperature (or for several days at 4°C) in a staining buffer containing sodium acetate (144 mM), sodium citrate (5.6 mM), copper sulfate (3.3 mM), potassium ferricyanide (0.54mM) and acetylthiocholine (2 mM) as a substrate for esterase enzymes. Stained specimens were mounted on 2% agarose pads and esterase localization scored on a Nikon E800 microscope. Color images were collected using a Hamamatsu ORCA-ER CCD camera with a LCI color filter using OpenLab software.

For lifespan assays, synchronized populations were collected by allowing gravid adult hermaphrodites to deposit eggs onto NGM plates containing OP50 bacteria or indicated RNAi bacteria. The embryos were allowed to hatch and develop to young adulthood at 15°C and young adults were transferred to fresh medium supplemented with 5-fluorodeoxyuracil (FUDR, 50 µg/mL final) to prevent progeny overgrowth of the plate. Lifespan assays were performed in a 20°C incubator, and animals were fed either OP50 or RNAi bacteria as a food source. For lifespan assays on RNAi bacteria, animals were transferred to new plates with a fresh RNAi bacterial lawn after one week (adult day 7).

For body length measurements, photographs were collected of young adult animals beginning after 96 hours of post-embryogenic development at 15°C. Body length was measured by measuring the length in pixels of a line tracing the distance from the animal's head to tail and converting from pixels to microns (108 pixels/100 microns for images collected using a 10× objective). Measurements were then standardized relative to length of *daf-2(e1370)* adults at each time point.

### Statistical analysis

Statistical analysis of lifespan assays was performed using JMP5.0 software package and statistical significance was judged using log-rank analysis. For body length measurements, statistical analysis was performed using Microsoft Excel 2004 and significance determined by students' t-test (2-tailed, unequal variance).

### Image analysis and processing

Animals were mounted on 2% agarose pads in M9 with levamisole as a paralyzing agent. Digital color and fluorescence images were collected using OpenLab software controlling a Hamamatsu Orca CCD camera. Image processing (cropping, rotating, etc.) was performed using Adobe Photoshop 7.0. For presentation purposes, some images were manipulated digitally to optimize the contrast and brightness. In these cases, all images presented within a figure were processed identically to maintain comparative differences. Image analysis was performed using functions within Image J (NIH Image) running on a Macintosh G4 computer. For quantification of *hsp-16.2*:GFP fluorescence, average pixel intensity in the pharynx corpus region was determined in adult animals. For background subtraction, average pixel intensity was measured in several same-sized regions of the microscope slide where no animals were present. The background intensity measurement determined from the average of these regions was subtracted from the average pixel intensity determined from the animals on that slide.
